# Strategic antagonism: how *Lactobacillus plantarum* counters *Staphylococcus aureus* pathogenicity

**DOI:** 10.3389/fmicb.2025.1635123

**Published:** 2025-08-04

**Authors:** Ly Tuan Kiet Bui, Fariha Alam Bushra, Pirachat Rattananon, Arifa Afrose Rimi, Carmen Lee, S. M. Tahmid, Sumaiya Akter Tisha, Irfan Fayaz Jisan, Rachita Das, Jahin Ibnat Sneha, Victoria Pitts, Olalekan Uchechukwu Owasanoye, Saphal Khadka, Shariful Islam

**Affiliations:** ^1^Department of Biological and Environmental Sciences, Southeast Missouri State University, Cape Girardeau, MO, United States; ^2^Biotechnology and Genetic Engineering Discipline, Khulna University, Khulna, Bangladesh

**Keywords:** *Staphylococcus aureus*, *Lactobacillus plantarum*, antagonism, probiotics, antimicrobial, microbiota, metabolite, antibiotic resistance

## Abstract

*Staphylococcus aureus* is a clinically significant pathogen known for its antibiotic resistance, immune evasion, and biofilm formation, making it a major contributor to persistent infections. *Lactobacillus plantarum*, a versatile probiotic bacterium, has emerged as a promising antagonist against *S. aureus* through multifaceted inhibitory mechanisms. This review synthesizes current evidence on the antagonistic interactions between *L. plantarum* and *S. aureus*, highlighting bacteriocin-mediated membrane disruption, quorum sensing interference, biofilm degradation, and metabolic competition. In addition, we explore how *L. plantarum* contributes to a less favorable inflammatory environment for *S. aureus* by modulating local immune responses at infection sites. Clinical relevance is explored across diverse anatomical sites such as the skin, vaginal tract, urinary system, and gastrointestinal tract, where *L. plantarum* demonstrates both direct and adjunctive therapeutic potential. We also consider environmental influences like pH and nutrient availability that modulate this antagonism. Together, the findings position *L. plantarum* as a compelling candidate for probiotic-based interventions against persistent and device-associated *S. aureus* infections.

## Introduction

*Staphylococcus aureus* has long been recognized as a notorious Gram-positive pathogen that invades skin, mucous membranes, and deeper tissues, leading to a wide range of clinical infections that pose significant challenges in healthcare settings (Tong et al., 2015). Its ability to form biofilms, evade host immune responses, and develop resistance to multiple antibiotics renders it as a formidable adversary, particularly in cases of device-associated infections and chronic wounds (Ahn et al., 2018; Brunel and Benoit, 2017; Otto, 2018). Due to this, considerable attention has been directed toward understanding the interactions between *S. aureus* and its host environment, with the aim of uncovering novel therapeutic strategies to combat its pathogenicity.

One promising area of research involves the utilization of probiotics, especially *Lactobacillus plantarum*, a species of bacteria that is part of the healthy gastrointestinal microbiota and has demonstrated to exhibit potent antimicrobial as well as anti-inflammatory properties (Douillard and de Vos, 2014; Hu et al., 2019; van den Nieuwboer et al., 2016). Studies have shown that *L. plantarum* can exert significant antagonistic effects against *S. aureus*, a dynamic that is evidenced by its multimodal ability to suppress the growth of *S. aureus* through the production of antimicrobial compounds and modulation of inflammatory responses (Ren et al., 2017). This antagonistic interplay not only highlights the potential of *L. plantarum* as a natural inhibitor of critical pathogens, but also opens avenues for its application as an adjunct or alternative to conventional antibiotic therapies (Brunel and Benoit, 2017; Hu et al., 2019; Dolan et al., 2017; Kalliomäki et al., 2003; Kalliomäki et al., 2007; Ong et al., 2020).

The clinical relevance of *L. plantarum* is further underscored by its role in specific host environments where *S. aureus* poses a significant risk. In the vaginal tract, for instance, *L. plantarum* naturally acidifies the local milieu through the production of lactic acid, thereby maintaining an environment that is hostile to pathogenic invaders. Moreover, engineered strains of *L. plantarum* expressing lysostaphin have been explored as innovative approaches to target *S. aureus* infections by degrading its cell wall components, which is particularly important in preventing conditions such as menstrual toxic shock syndrome (Boskey et al., 2001; Boskey et al., 1999; Liu et al., 2011; Sodré et al., 2023; Arena et al., 2016; Li et al., 2023a; Turner et al., 2007). In addition to the vaginal ecosystem, the probiotic potential of *L. plantarum* has been implicated in enhancing wound healing and modulating immune responses at other anatomical sites, thereby contributing to a reduction in the incidence and severity of *S. aureus* infections. Recent studies have provided compelling evidence that the ability of *L. plantarum* to interfere with biofilm formation and disrupt established biofilms could serve as a crucial therapeutic strategy in overcoming persistent infections (Reid et al., 2001; Rönnqvist et al., 2006; Wang et al., 2018; Wang et al., 2021). This review seeks to synthesize the current understanding of the interactions between *L. plantarum* and *S. aureus*, focusing on the underlying mechanisms of antagonisms, its clinical implications, and the potential for developing probiotic-based interventions to address the challenges posed by *S. aureus* in diverse clinical scenarios.

By integrating findings from *in vitro* studies, mammalian models, and clinical investigations, this review aims to provide a comprehensive overview of the role of *L. plantarum* as a therapeutic agent in the management of *S. aureus* infections. This serves to lay the groundwork for future research and to inform clinical practice by highlighting both the benefits and limitations of leveraging probiotic strategies such as *L. plantarum* against *S. aureus* that continues to challenge conventional therapeutic paradigms.

## Mechanisms of *Lactobacillus plantarum* antagonism against *Staphylococcus aureus*

*L. plantarum* counters *S. aureus* through several distinct yet complementary mechanisms. These include the secretion of bacteriocins that disrupt membrane integrity, interference with *S. aureus* quorum sensing pathways that regulate virulence, and modulation of host immune responses to reduce inflammation and promote pathogen clearance. This section outlines these strategies in detail, highlighting the multifaceted nature of *L. plantarum*’s antagonism.

**Figure 1:**
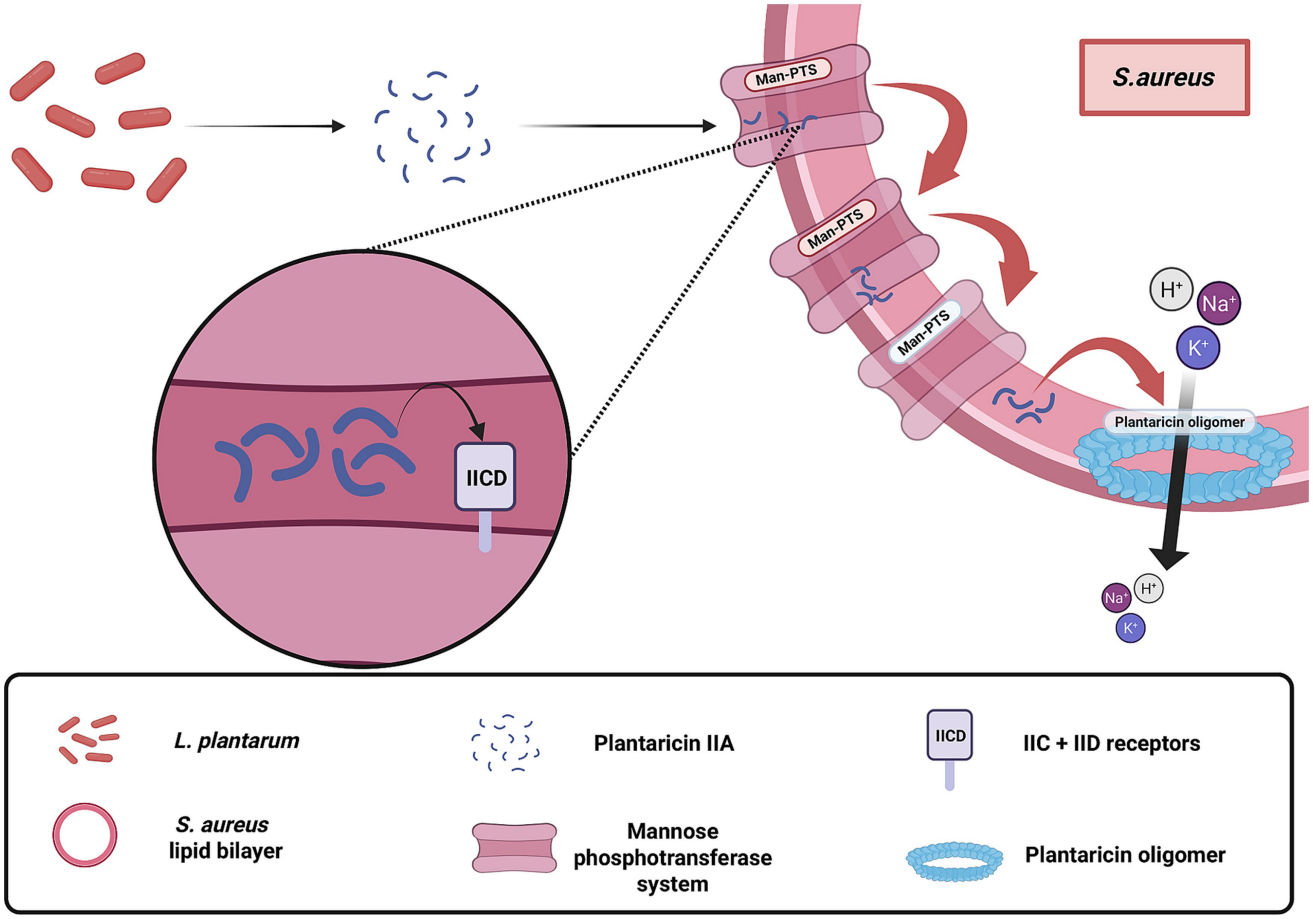
Membrane-disruptive mechanism of plantaricin-class bacteriocins produced by *Lactobacillus plantarum* targeting *Staphylococcus aureus*. These bacteriocins, primarily from the plantaricin family, exert direct antimicrobial activity by embedding into the bacterial phospholipid bilayer, oligomerizing, and forming pores that lead to ion leakage and cell death. The figure illustrates the receptor-dependent action of class IIA plantaricins, such as plantaricin IIA-1A5, which utilize the Mannose Phosphotransferase System, particularly the IIC and IID subunits, as docking sites for membrane localization and insertion. This mode of action contributes to the direct killing of *S. aureus* independent of host factors. Created with BioRender.

One principal mechanism involves the production and secretion of bacteriocins, proteinaceous molecules that directly target *S. aureus* by compromising cell membrane integrity and inhibiting biofilm formation (Sodré et al., 2023; Arena et al., 2016). Although the exact mechanisms differ among bacteriocin subclasses, most plantaricins ultimately disrupt the target membrane, leading to ion leakage and cell death. For instance, class IIA plantaricins specifically target the mannose phosphotransferase system (Man-PTS) in *S. aureus* (Figure 1), with the IIC and IID subunits serving as docking sites for insertion into the lipid bilayer (Arief et al., 2015; Tymoszewska et al., 2018; Oppegård et al., 2016). Upon localization, they infiltrate the bacterial phospholipid bilayer and form oligomers, which disrupt the regularity of the structure and induce pore formation. This puncture leads to the loss of cytosolic components, most notably the electrolytic leakage of ions such as potassium and sodium, as well as amino acids and other solutes (Ennahar et al., 2000). In a similar vein, plantaricin YKX interferes with the ionic homeostasis of *S. aureus* by destabilizing cell signaling pathways that are critical for biofilm formation (Pei et al., 2022). Additionally, certain bacteriocins such as plantacyclin B21AG are characterized by a circular structure, which endows them with enhanced resistance to proteolytic enzymes, thereby ensuring sustained antimicrobial activity even in diverse and challenging environments (Golneshin et al., 2020). These bacteriocin-mediated effects not only curtail the proliferation of *S. aureus* but also contribute to reducing the likelihood of resistance development.

Beyond direct membrane disruption, *Lactiplantibacillus* species can interfere with intercellular communication systems, particularly the quorum sensing system in *S. aureus*, which regulates virulence and biofilm formation. Among these species, *Lactiplantibacillus paraplantarum* shows a strong ability to inhibit *S. aureus* quorum sensing by producing AIP-like peptides through its *agr*-like *lam*BDCA system. These peptides can suppress *S. aureus agr* activation, especially in *agr* groups I, II, and IV, leading to significant reductions in hemolysin production and virulence gene expression (Wang et al., 2025). The inhibitory effect is context dependent, with stronger suppression observed in co-culture conditions compared to cell-free supernatants. Disruption of the *lam*BDCA locus eliminates this effect, confirming its essential role. *L. plantarum* also contains a *lam*BDCA locus and produces an AIP peptide known as LamD558, but its inhibitory effects on *S. aureus* are modest and mainly limited to *agr* group I (Wang et al., 2025). Structural differences in the peptide, including its tailless thiolactone conformation, may influence its stability and activity. Additionally, other small molecules such as dipeptides like cyclo (L-Phe-L-Pro) may contribute to the limited quorum sensing interference observed in *L. plantarum.* Collectively, these disruptions weaken *S. aureus* coordination of adhesion, toxin production, and biofilm formation (Arena et al., 2016; Wang et al., 2025; Qazi et al., 2006; Spangler et al., 2019).

**Figure 2:**
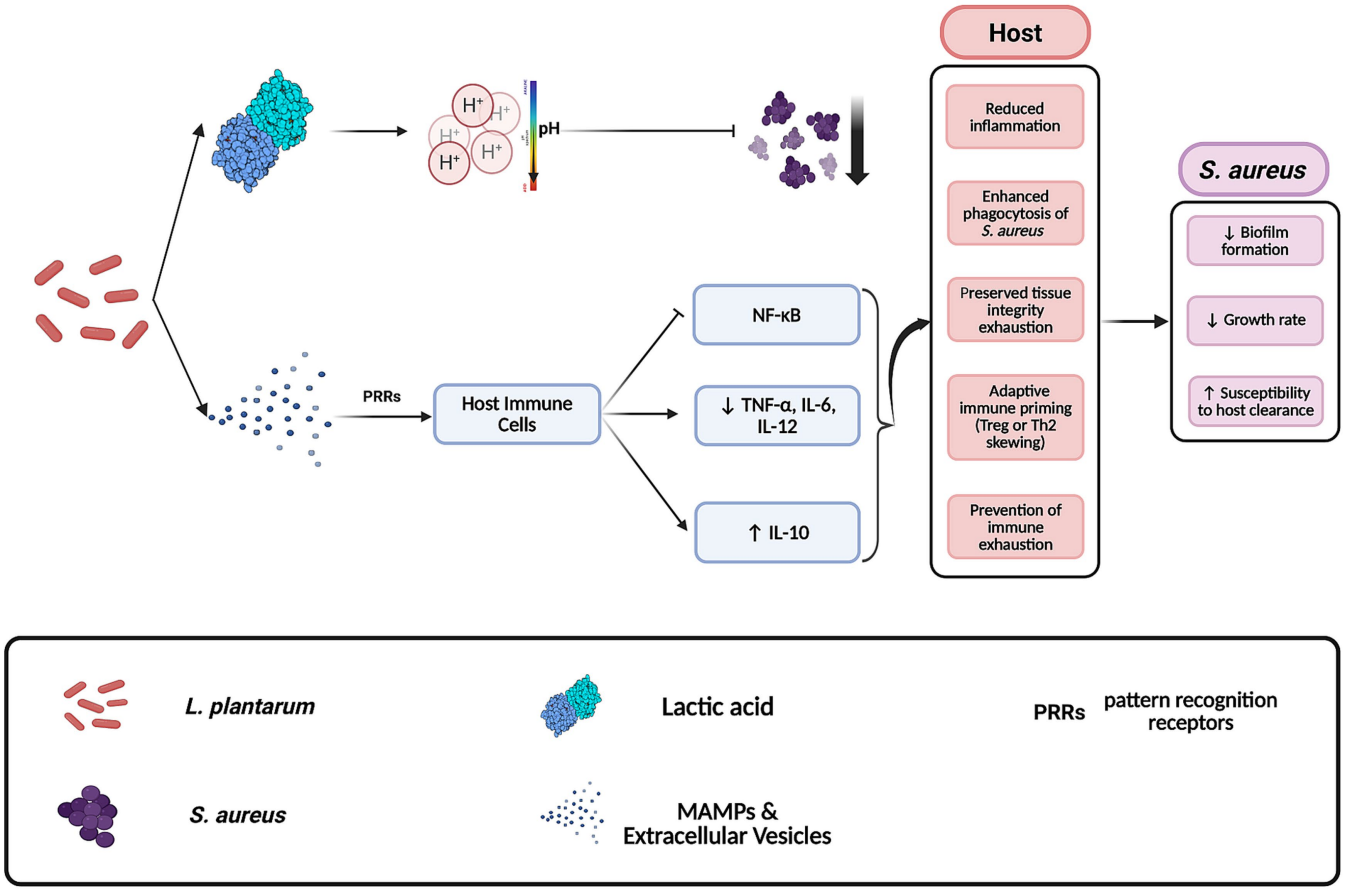
Mechanisms by which *Lactobacillus plantarum* antagonizes *Staphylococcus aureus* in host environments. *L. plantarum* inhibits *S. aureus* through the secretion of antimicrobial compounds such as lactic acid and by modulating host immune responses. This includes the release of extracellular vesicles that contribute to anti-inflammatory signaling in host tissues. These combined effects reduce *S. aureus* virulence and proliferation. Created with BioRender.

In addition to bacterial interactions, *L. plantarum* also influences the host immune response through interactions with their innate and adaptive systems (Ong et al., 2020), facilitating indirect counteraction against *S. aureus* pathogenesis (Figure 2). Recent studies have demonstrated that components of *L. plantarum* are recognized by host immune cells and mediate the outcomes of reduced site inflammation (Ong et al., 2020). Wound-healing is shown to be preserved in the host due to a mixture of bioactive components, which include both nanoparticles and small metabolites, derived from lysates of *L. plantarum* strain K8 (K8NPs) for both *in vivo* and *in vitro* murine models (Ong et al., 2020; Hong et al., 2023). In live cells, these molecules are exported through extracellular vesicles (Kim et al., 2018). One known element, lipoteichoic acid, binds with TLR2 and TLR6 downregulating the NF-kB pathway (Wells, 2011). This reduction in pro-inflammatory mediators not only diminishes tissue damage during *S. aureus* infections but also enhances the overall capacity of the host’s immune system to combat the pathogenesis (Hong et al., 2023). Together, these effects position *L. plantarum* as a potent antagonist capable of targeting both the pathogen and the inflammatory environment it exploits.

## *Staphylococcus aureus* biofilm formation and *Lactobacillus plantarum* interventions

*Staphylococcus aureus* is well recognized for its capacity to form robust biofilms, which serve as a formidable defense mechanism against both antibiotic treatments and host immune responses. These biofilms are complex structures composed of exopolysaccharides, proteins, and extracellular DNA that envelop bacterial cells, thereby protecting them from environmental stressors and facilitating their persistence on medical devices and tissues (Otto, 2018; Jefferson, 2004). Biofilm development occurs in several distinct stages: initially, free-floating bacterial cells adhere to surfaces and aggregate to form microcolonies. These clusters then produce a sticky extracellular matrix (ECM) that binds the cells together into a three-dimensional structure. Finally, when the biofilm reaches a critical density, signaling prompt partial disintegration of the ECM, allowing some bacteria to disperse and colonize new niches (Moormeier and Bayles, 2017). This dynamic process not only contributes to the chronicity of *S. aureus* infections but also complicates their treatment, particularly in clinical settings where biofilm-associated infections are notoriously resistant to conventional antibiotics.

**Figure 3:**
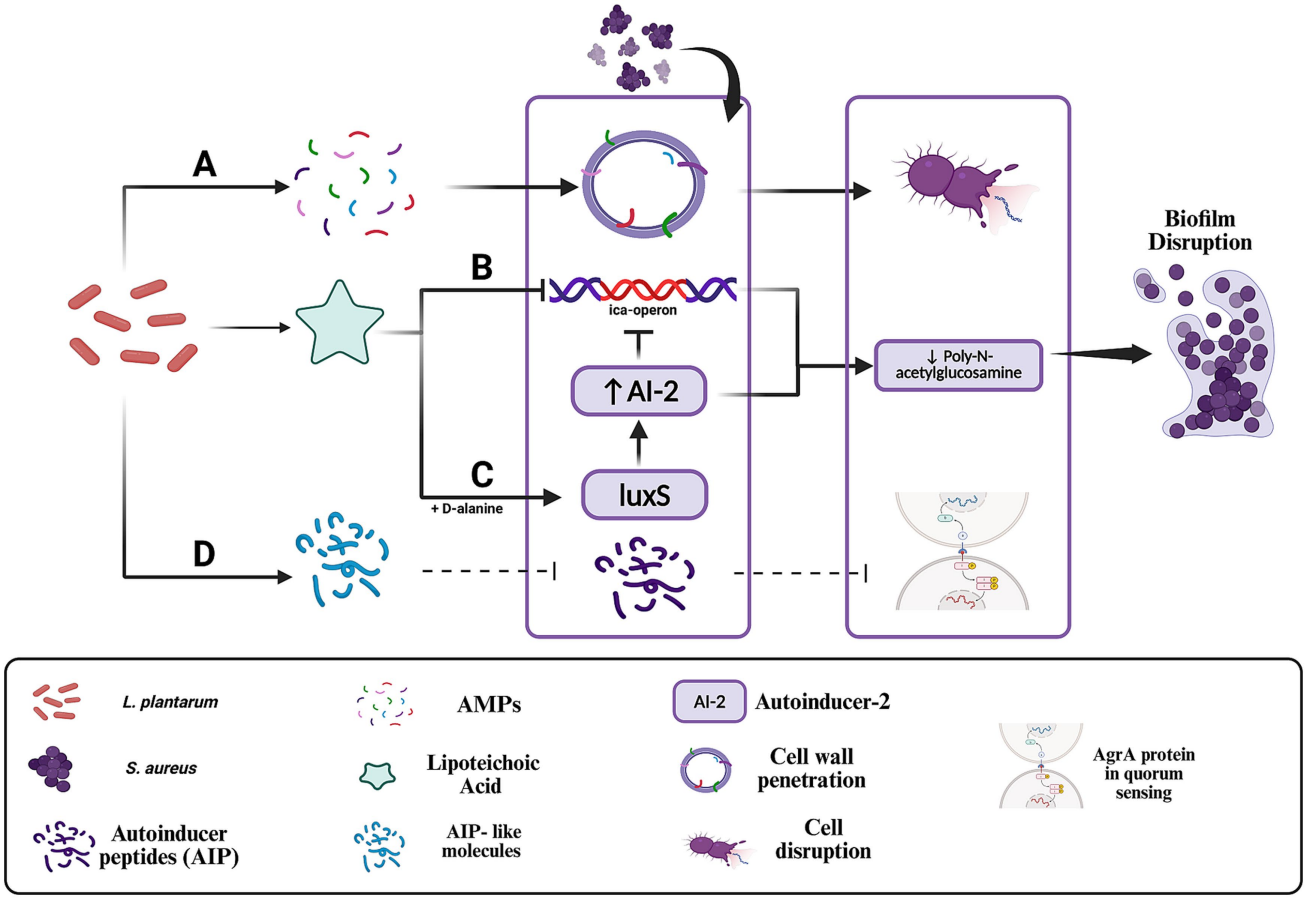
Mechanisms by which *Lactobacillus plantarum* disrupts *Staphylococcus aureus* quorum sensing and biofilm formation. **(A)** Antimicrobial peptides (AMPs) disrupt the bacterial membrane and inhibit the synthesis of essential biomolecules, leading to cell lysis. **(B)** Lipoteichoic acid (LTA) inhibits the ica operon, leading to reduced synthesis of poly-N-acetylglucosamine, a major structural component of the biofilm extracellular matrix. **(C)** D-alanine-modified LTA enhances *luxS* expression, increasing autoinducer-2 production and disrupting quorum-regulated gene expression critical for biofilm maturation. **(D)** AIP-like molecules produced by *L. plantarum* may competitively bind to the *Agr*C receptor, interfering with *S. aureus* quorum sensing. In engineered strains, secreted proteases such as lysostaphin may further degrade AIPs, amplifying quorum disruption. Created with BioRender.

In response to the protective biofilm formation of *S. aureus*, *L. plantarum* employs a suite of targeted interventions aimed at both preventing biofilm development and disrupting established biofilms (Figure 3). One of the primary mechanisms involves the secretion of lipoteichoic acid (LTA) (Ahn et al., 2018). *L. plantarum* releases LTA that actively inhibits biofilm formation by suppressing the expression of the *ica*-operon, a critical genetic locus responsible for the synthesis of poly-*N*-acetylglucosamine (Figure 3B), a key component of the biofilm matrix (Ahn et al., 2018; Yasir et al., 2018).

This inhibitory effect is further enhanced by the LTA-induced release of autoinducer-2. However, LTA lacking D-alanine moieties is ineffective at influencing the *luxS* gene in *S. aureus* and fails to upregulate autoinducer-2 (Ahn et al., 2018). Through this mechanism, *L. plantarum* disrupts the ability of *S. aureus* to organize into a cohesive and protective biofilm structure (Figure 3C). In addition to LTA, *L. plantarum* produces antimicrobial peptides (AMPs) (Figure 3A) that contribute to the disintegration of biofilms. These AMPs interact with the lipoteichoic acid in the cell walls of Gram-positive bacteria, such as *S. aureus*, and penetrate the bacterial membrane, leading to cell lysis. The action of these peptides extends beyond mere membrane disruption; they also inhibit the synthesis of essential cellular components, including cell wall, DNA, RNA, and proteins, and can trigger autolytic enzyme activity, thereby promoting bacterial self-destruction (Yasir et al., 2018). This multifaceted approach not only reduces the viability of individual bacterial cells but also compromises the overall integrity of the biofilm matrix.

Another important strategy employed by *L. plantarum* is the interference with quorum-sensing mechanisms that *S. aureus* relies upon for biofilm maturation and maintenance. Quorum sensing is a critical communication system that coordinates the expression of virulence factors and biofilm-associated genes. *L. plantarum* disrupts this process by secreting organic acids and producing signaling molecules that interfere with *S. aureus* quorum sensing (Figure 3D) (Arena et al., 2016; Wang et al., 2025; Li et al., 2023b). Adapting from this approach, engineered strains of *L. plantarum* have been developed to sense *S. aureus* quorum-sensing signals and respond by producing lysostaphin, a potent bacteriolytic enzyme that specifically targets and degrades the cell wall of *S. aureus*, which prevent biofilm formation (Li et al., 2023a; Turner et al., 2007). This targeted interference with quorum sensing represents a promising avenue for mitigating the coordinated defense mechanisms of *S. aureus*. Beyond these molecular strategies, *L. plantarum* also secretes various matrix-degrading enzymes, including biosurfactants and proteases, which play a crucial role in dismantling the structural integrity of microorganism biofilms. One paper has investigated these substances in *Pseudomonas aeruginosa* where they act to degrade the extracellular matrix, thereby increasing the susceptibility of the embedded bacteria to both the host’s immune defenses and antimicrobial agents (Li et al., 2023b). Additionally, the production of lactic acid by *L. plantarum* not only contributes to a lower pH that destabilizes the biofilm but also directly inhibits microbial growth, further diminishing the survival prospects of *S. aureus* within its protective niche (Tejero-Sariñena et al., 2012). Research has also demonstrated that *L. plantarum* can secrete bacteriocins and exopolysaccharides which further disrupt biofilm production, and that the bacterium is capable of disintegrating existing biofilms through the action of proteolytic enzymes, effectively reducing biofilm viability when co-cultured with *S. aureus* (Arena et al., 2016; Vuotto et al., 2014).

## Clinical applications in specific infection sites

**Figure 4:**
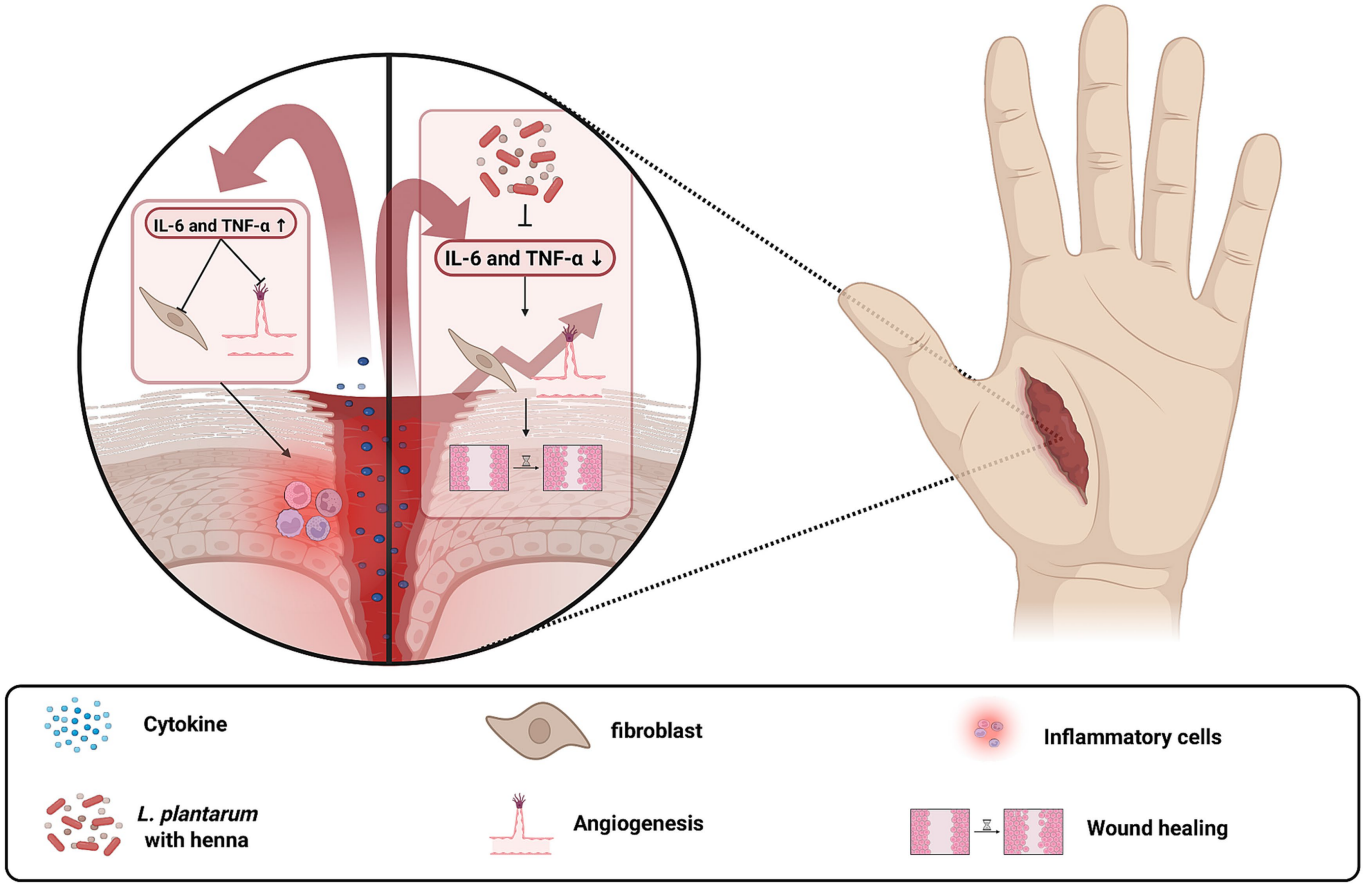
Inhibition of IL-6 and TNF-α promotes wound healing by reducing excessive inflammation and enhancing tissue regeneration. The left panel illustrates a condition with high IL-6 and TNF-α levels, leading to prolonged inflammation, impaired fibroblast activity, and delayed wound closure. The right panel shows the effects of IL-6 and TNF-α inhibition by *L. plantarum* with *Lawsonia inermis*, which reduces bacterial load, enhances fibroblast proliferation, promotes collagen deposition, and accelerates wound healing. Created with BioRender.

The clinical utility of *L. plantarum* in countering *S. aureus* infections has been demonstrated across a variety of anatomical sites, underscoring its potential as both a standalone therapeutic and an adjunct to conventional antibiotic treatments (Ong et al., 2020; Mukherjee and Ramesh, 2015; Rizzo et al., 2015). In the context of skin infections, especially those involving wounds, the combination of *L. plantarum* with *Lawsonia inermis* (henna) has shown promising results. Studies indicate that this combination effectively reduces inflammatory cytokines such as interleukin (IL)-6 and tumor necrosis factor (TNF)-α, thereby accelerating the healing process in *S. aureus*-infected wounds (Elebeedy et al., 2022). Excessive inflammation is a major barrier to efficient wound healing, and pro-inflammatory cytokines like IL-1, IL-6 and TNF-α play critical roles in this process (Figure 4) (Kagiwada et al., 2004). While these cytokines are essential for early immune responses, their prolonged activity inhibits fibroblast proliferation, delays collagen deposition, and impairs angiogenesis, ultimately slowing tissue repair (Barrientos et al., 2008; Eming et al., 2007). Inhibition of IL-6 and TNF-α promotes a faster transition from the inflammatory phase to the proliferative phase, reducing oxidative stress and enhancing re-epithelialization, which is particularly beneficial for chronic wounds (Eming et al., 2007). This synergistic effect not only enhances bacterial clearance but also mitigates local inflammation, which is a critical factor in managing infections that are often complicated by antibiotic resistance.

The vaginal tract represents another important site where *L. plantarum* exhibits significant clinical benefits (Reid et al., 2001; Rönnqvist et al., 2006; Spurbeck and Arvidson, 2011). Naturally, *L. plantarum* produces lactic acid, which maintains an acidic environment that suppresses the growth of *S. aureus*. Furthermore, the presence of *L. plantarum* prevent the adhesive proteins of *S. aureus* from attaching to the epithelial surfaces of the vaginal tract, thereby reducing the likelihood of colonization and subsequent infection (Christensen et al., 2021). Lysostaphin in the engineered strain also aids in this process by degrading cell walls and preventing biofilm formation (Liu et al., 2011; Turner et al., 2007; Christensen et al., 2021). This dual approach of environmental acidification and targeted enzymatic action is particularly valuable in preventing conditions such as toxic shock syndrome and other *S. aureus*-associated vaginal infections.

**Figure 5:**
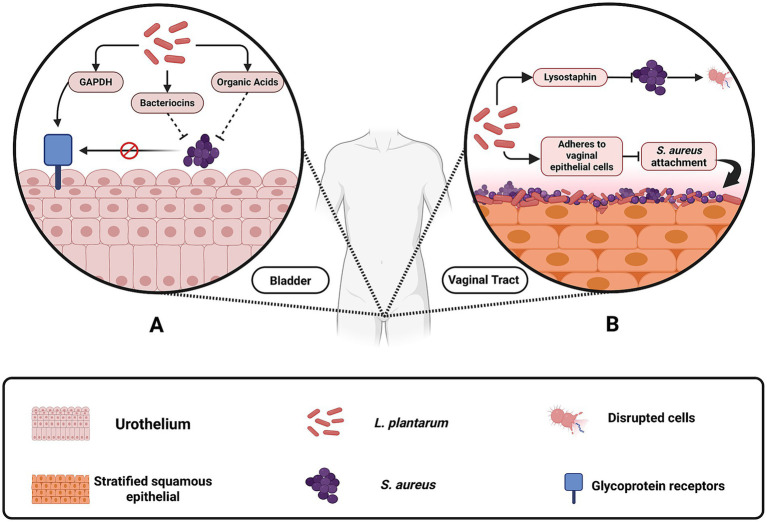
Mechanisms by which *Lactobacillus plantarum* inhibits *Staphylococcus aureus* colonization in the vaginal and urinary tracts. **(A)** In the urinary tract, *L. plantarum* produces glyceraldehyde-3-phosphate dehydrogenase (GAPDH), bacteriocins, and organic acids, which prevent *S. aureus* from adhering to bladder epithelial cells and forming biofilms on urinary devices. **(B)** In the vaginal tract, *L. plantarum* contributes to acidification by releasing organic acids, which lower pH and suppress *S. aureus* growth. Additionally, engineered *L. plantarum* strains produce lysostaphin, an enzyme that degrades the *S. aureus* cell wall, and adhere to vaginal epithelial cells, blocking *S. aureus* attachment and colonization. These protective mechanisms reduce the risk of infections such as toxic shock syndrome and catheter-associated urinary tract infections. Created with BioRender.

Urinary tract infections, particularly those related to indwelling medical devices, present another clinical challenge where *L. plantarum* has demonstrated potential benefits. Although *S. aureus* is responsible for only a small percentage of urinary tract infections, its capacity to form biofilms on urinary devices such as catheters and stents leads to persistent and often difficult-to-treat infections (Carvalho et al., 2021; Klein and Hultgren, 2020; Walker et al., 2017). *L. plantarum* can effectively compete with *S. aureus* by adhering to bladder epithelial cells, a process mediated by proteins such as glyceraldehyde-3-phosphate dehydrogenase (GAPDH) (Wang et al., 2018; Wang et al., 2021). While GAPDH is well known as a key enzyme in glycolysis, in *L. plantarum* it also “moonlights” as a cell surface adhesin. Once translocated to the bacterial surface via non-classical secretion pathways, GAPDH binds to glycoprotein receptors on bladder epithelial cells (Figure 5) through conserved amino acid motifs (Wang et al., 2018; Reid and Tieszer, 1994; Wang et al., 2022). Furthermore, its secretion of antimicrobial agents including plantaricin, lactic acid and hydrogen peroxide contributes to the disruption of *S. aureus* biofilms. This integrated mechanism, combining competitive adhesion with targeted antimicrobial production, reinforces *L. plantarum*’s capacity to prevent *S. aureus* colonization on urinary devices.

**Figure 6:**
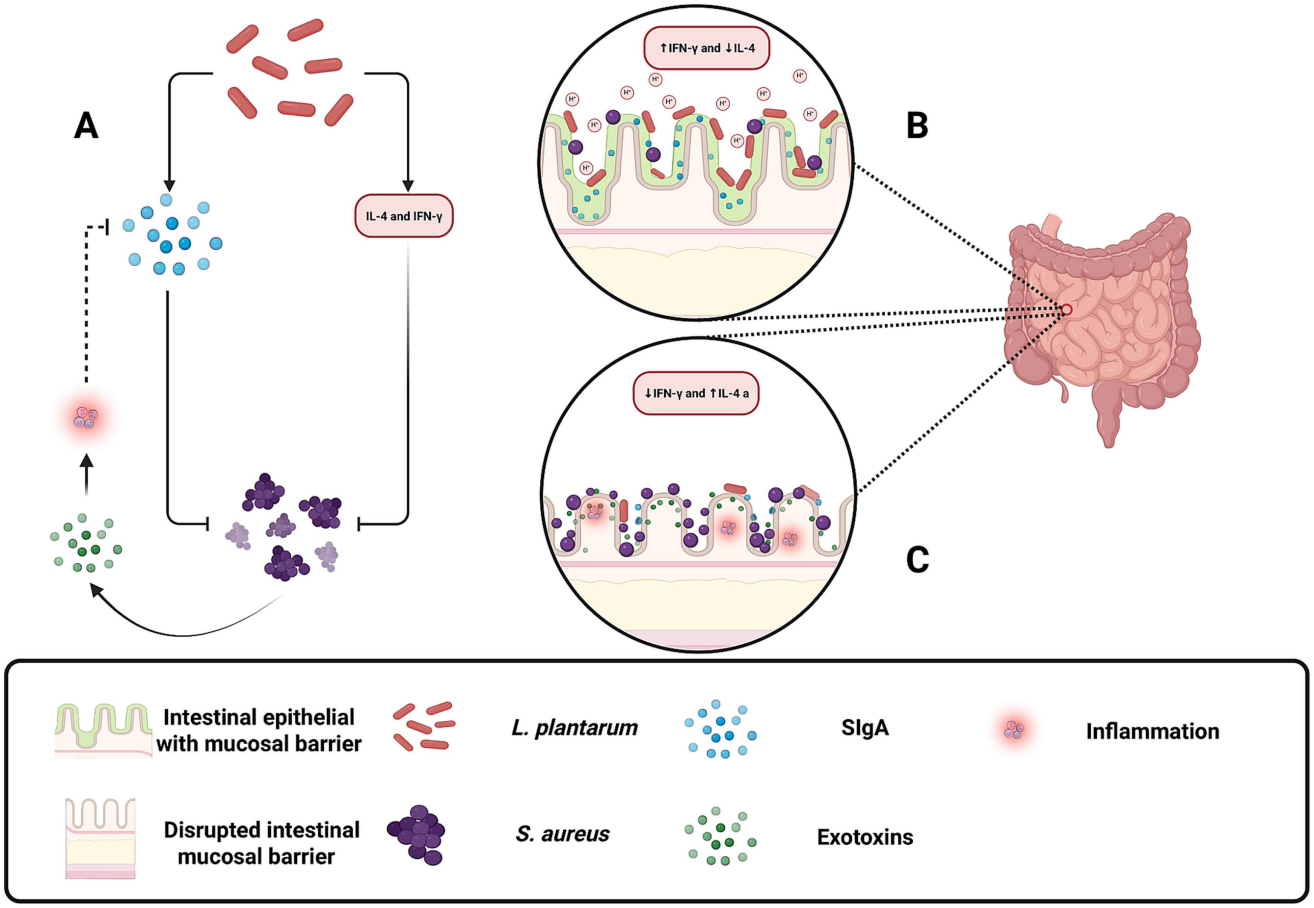
Protective role of *Lactobacillus plantarum* in maintaining gut homeostasis and preventing *Staphylococcus aureus* pathogenesis. **(A)** Illustrates how *L. plantarum* indirectly attenuates inflammation by promoting SIgA secretion and reducing *S. aureus* exotoxin-induced immune activation. **(B)** *L. plantarum* helps restore gut balance by producing lactic acid, lowering gut pH to inhibit *S. aureus* growth, and modulating the immune response by increasing IFN-γ and decreasing IL-4 levels. Additionally, *L. plantarum* enhances SIgA secretion, strengthening the mucosal barrier and preventing *S. aureus* adhesion and infection. **(C)** Under disrupted conditions, *S. aureus* overgrows, releases exotoxins, and degrades secretory immunoglobulin A (SIgA), leading to inflammation and epithelial damage. Increased IL-4 and reduced IFN-γ levels contribute to immune dysregulation and pathogen invasion. Created with BioRender.

Within the gastrointestinal tract, *L. plantarum* plays a vital role in maintaining gut homeostasis and preventing the transition of *S. aureus* from a commensal organism to a pathogen. Although *S. aureus* may be present as part of the normal gut flora, disruptions in intestinal homeostasis or immune function can trigger its pathogenic potential, leading to the release of exotoxins and degradation of secretory immunoglobulin A (SIgA) (Ren et al., 2017). *L. plantarum* contributes to intestinal health by producing lactic acid, which lowers the pH and inhibits *S. aureus* growth (Figure 2), while also modulating the immune response by enhancing interferon-gamma (IFN)-γ production and reducing IL-4 levels (Figure 6) (Ren et al., 2017; Rizzo et al., 2015; Lin et al., 2013). Additionally, it stimulates SIgA secretion, thereby strengthening the mucosal barrier and preventing pathogen invasion (Ren et al., 2017; Rizzo et al., 2015; Lin et al., 2013). One article also reported that *Lactobacillus plantarum* extracts inhibited HT-29 colon cancer cell apoptosis induced by *Staphylococcus aureus* and its alpha-toxin, demonstrating a potential role for probiotic-derived compounds in mitigating the harmful effects of pathogenic bacteria and their toxins on intestinal epithelial cells (Kim et al., 2015). This multifaceted probiotic activity helps to preserve the delicate balance of the gut microbiota and protects against inflammation and infection (Wang et al., 2024).

## Environmental influences on *Lactobacillus plantarum* and *Staphylococcus aureus* interactions

Environmental factors such as pH, temperature, and nutrient availability play a pivotal role in modulating the interactions between *Lactobacillus plantarum* and *Staphylococcus aureus*. Among these, temperature is a key determinant of *L. plantarum*’s metabolic activity, with optimal production of lactic acid and bacteriocins observed between 30°C and 37°C (Sodré et al., 2023; Arena et al., 2016; Golneshin et al., 2020). These antimicrobial products contribute to environmental acidification, which disrupts *S. aureus* metabolism, inhibits biofilm formation, and impairs expression of virulence genes sensitive to low pH conditions.

Another critical environmental pressure is oxidative stress, particularly in host-associated niches where reactive oxygen species (ROS) are abundant. While *L. plantarum* does not actively produce hydrogen peroxide in significant quantities, it demonstrates notable tolerance to oxidative environments. This resilience is partly attributed to its ability to utilize exogenous quinones for extracellular electron transport, a process that helps maintain redox balance and energy production under stress conditions (Stevens et al., 2023; Tian et al., 2022; Lebeer et al., 2008). Additionally, *L. plantarum* has been shown to attenuate host inflammation in *S. aureus*-infected tissues, likely reducing oxidative bursts through immune modulation (Xie et al., 2025). These findings suggest that *L. plantarum* may gain a competitive advantage not by generating oxidative stress, but by withstanding and adapting to it more effectively than *S. aureus* (Kondakala et al., 2024).

Beyond stress tolerance, nutrient availability is another crucial environmental factor influencing the competitive dynamics between these two microorganisms. *L. plantarum* is endowed with remarkable metabolic versatility, which enables it to utilize a wide array of sugars and amino acids efficiently. This versatility is facilitated by specialized sugar transport systems, such as sacPTS1 and sacPTS26, which allow *L. plantarum* to metabolize complex carbohydrates like fructooligosaccharides (FOS) and sucrose even under carbon-limited conditions (Cui et al., 2021). In contrast, *S. aureus* primarily depends on simpler carbon sources such as glucose and lactate. The relatively narrow metabolic repertoire of *S. aureus* renders it less competitive in nutrient-deprived environments compared to the adaptable *L. plantarum* (Halsey et al., 2017).

Furthermore, nitrogen acquisition represents a critical aspect of this competitive interplay. *S. aureus* relies on the catabolism of specific amino acids particularly those derived from proline, arginine, and histidine to fulfill its nitrogen requirements. This process is tightly regulated by systems such as CodY and Carbon Catabolite Repression (CCR), which prioritize amino acid utilization based on nutrient availability (Halsey et al., 2017; Somerville and Proctor, 2009). In contrast, *L. plantarum* employs proteolytic mechanisms, including the oligopeptide permease (Opp) system, to break down peptides into amino acids, ensuring a steady and efficient nitrogen supply (Halsey et al., 2017; Hu et al., 2024; Rezaei et al., 2021). Additionally, *L. plantarum* benefits from cross-feeding mechanisms in co-culture environments, utilizing metabolic byproducts released by other microorganisms. This strategy not only minimizes direct competition with *S. aureus* but also enhances the survival and persistence of *L. plantarum* in nitrogen-limited conditions (Hu et al., 2024; Rezaei et al., 2021).

Together, these environmental influences, including optimal temperature, fostering robust antimicrobial production and versatile nutrient utilization create conditions that favor the dominance of *L. plantarum* over *S. aureus* (Halsey et al., 2017; Somerville and Proctor, 2009; Hu et al., 2024; Rezaei et al., 2021). By thriving in environments that are less favorable to *S. aureus*, *L. plantarum* is better positioned to exert its probiotic and therapeutic effects, thereby contributing to the control of *S. aureus* infections in clinical settings.

## Conclusion

*Lactobacillus plantarum* antagonism against *Staphylococcus aureus* has emerged as an area of growing interest, offering promising therapeutic strategies for managing biofilm-associated and antimicrobial-resistant infections. Importantly, the antimicrobial potential of *L. plantarum* is strain-dependent. Comparative genomic analyses have revealed substantial phylogenetic and functional diversity within the species, particularly in bacteriocin gene clusters, stress response elements, and quorum sensing systems (Choi et al., 2021). This strain-specific behavior is further supported by studies showing that plantaricin A requires precise peptide cooperation for activity (Nissen-Meyer et al., 1993), and that plantaricin gene activation is regulated in response to co-culture with specific Gram-positive bacteria (Maldonado et al., 2004). This diversity underscores the importance of targeted, strain-level evaluation when considering *L. plantarum* as a therapeutic agent.

This review further synthesizes current findings on the diverse inhibitory strategies used by *L. plantarum*, including bacteriocin production, metabolic acidification, quorum sensing interference, and immune modulation. These mechanisms collectively position *L. plantarum* as a compelling adjunct or alternative to conventional antibiotic therapies in the face of rising resistance (Marianelli et al., 2010). By interfering with cell wall synthesis, quorum sensing, and biofilm formation, *L. plantarum* effectively suppresses *S. aureus* growth and virulence (Ahn et al., 2018; Arena et al., 2016; Yasir et al., 2018; Wang et al., 2022), suggesting that probiotic–pathogen interactions could drive a paradigm shift in antimicrobial strategies beyond traditional antibiotics. Given the prevalence of recurrent *S. aureus* infections in clinical contexts, particularly in wound care, leveraging *L. plantarum* as a therapeutic probiotic merits further exploration. Its potential applications include prevention of medical-device-associated infections and modulation of mucosal microbiota (Ong et al., 2020). Nevertheless, significant challenges remain in translating laboratory findings into effective clinical interventions. Future directions should include comprehensive phylogenetic profiling, elucidation of molecular mechanisms underpinning probiotic–pathogen interactions, and well-controlled clinical trials with matched populations to rigorously assess the efficacy and safety of *L. plantarum*-based therapies (Aljohani et al., 2024; Parvez et al., 2006).
